# Interesterified Palm Olein (IEPalm) and Interesterified Stearic Acid-Rich Fat Blend (IEStear) Have No Adverse Effects on Insulin Resistance: A Randomized Control Trial

**DOI:** 10.3390/nu10081112

**Published:** 2018-08-17

**Authors:** Yen Teng Ng, Phooi Tee Voon, Tony Kock Wai Ng, Verna Kar Mun Lee, Miskandar Mat Sahri, Norhaizan Mohd Esa, Seng Huat Ong, Augustine Soon Hock Ong

**Affiliations:** 1 Department of Nutrition and Dietetics, School of Health Sciences, International Medical University, Bukit Jalil, 57000 Kuala Lumpur, Malaysia; ngyenteng.mpob@gmail.com; 2 Nutrition Unit, Malaysian Palm Oil Board, 6, Persiaran Institusi, Bandar Baru Bangi, 43000 Kajang, Selangor, Malaysia; 3 Department of Biomedical Sciences, Faculty of Science, Universiti Tunku Abdul Rahman (UTAR), 31900 Kampar, Perak, Malaysia; drtonyngkw@gmail.com or ngkw@utar.edu.my; 4 Department of Family Medicine, School of Medicine, International Medical University, Clinical Campus, 70300 Seremban, Negeri Sembilan, Malaysia; a_lee@imu.edu.my or verna_lee@imu.edu.my; 5 Protein and Food Technology Unit, Malaysian Palm Oil Board, 6, Persiaran Institusi, Bandar Baru Bangi, 43000 Kajang, Selangor, Malaysia; miskand@mpob.gov.my; 6 Department of Nutrition and Dietetics, Faculty of Medicine and Health Sciences, Universiti Putra Malaysia, 43400 Serdang, Selangor, Malaysia; nhaizan@upm.edu.my; 7 Department of Actuarial Science and Applied Statistics, UCSI University, 56000 Kuala Lumpur, Malaysia; ongsh@um.edu.my or ongsh@usciuniversity.edu.my; 8 Academy of Sciences, Malaysia, 47400 Petaling Jaya, Selangor, Malaysia; tansriong@yahoo.com

**Keywords:** interesterified fats, palm-based margarine, insulin resistance, serum lipid, clinical human feeding trial

## Abstract

Chemically-interesterified (CIE) fats are *trans*-fat free and are increasingly being used as an alternative to hydrogenated oils for food manufacturing industries to optimize their products’ characteristics and nutrient compositions. The metabolic effects of CIE fats on insulin activity, lipids, and adiposity in humans are not well established. We investigated the effects of CIE fats rich in palmitic (C16:0, IEPalm) and stearic (C18:0, IEStear) acids on insulin resistance, serum lipids, apolipoprotein concentrations, and adiposity, using C16:0-rich natural palm olein (NatPO) as the control. We designed a parallel, double-blind clinical trial. Three test fats were used to prepare daily snacks for consumption with a standard background diet over a period of 8 weeks by three groups of a total of 85 healthy, overweight adult volunteers. We measured the outcome variables at weeks 0, 6, and at the endpoint of 8. After 8 weeks, there was no significant difference in surrogate biomarkers of insulin resistance in any of the IE fat diets (IEPalm and IEStear) compared to the NatPO diet. The change in serum triacylglycerol concentrations was significantly lower with the IEStear diet, and the changes in serum leptin and body fat percentages were significantly lower in the NatPO-diet compared to the IEPalm diet. We conclude that diets containing C16:0 and C18:0-rich CIE fats do not affect markers of insulin resistance compared to a natural C16:0-rich fat (NatPO) diet. Higher amounts of saturated fatty acids (SFAs) and longer chain SFAs situated at the *sn*-1,3 position of the triacylglycerol (TAG) backbones resulted in less weight gain and lower changes in body fat percentage and leptin concentration to those observed in NatPO and IEStear.

## 1. Introduction

Liquid vegetable oils are traditionally solidified or “hardened” for household use by partial hydrogenation at high temperatures—a process which produces substantial amounts of *trans* fatty acids (TFAs) which raises serum lipids as well as being atherogenic [1,2]. With the goal of producing *trans*-free food products, modified fats other than partial hydrogenated fats are utilized in food products to optimize their characteristics and nutrient compositions. This can be achieved by blending, fractionation, interesterification, or full hydrogenation.

Nowadays, the interesterification process is commonly used as an alternative to hydrogenation to produce hardened fats without the TFAs. Palm-based oils and interesterified fats (IE) fats made with fully hydrogenated soybean oil have been used as alternatives to replace TFA-containing oils in foods, such as shortening, margarine, and confectionary products [3]. By referring to National Health and Nutrition Examination Survey (NHANES) 1992–2002 intake data, 25 food categories were identified, representing 86% of total soybean oil intake and 79% of total TFA intake in the United States [4,5]. Among the 25 food categories that include TFA-containing oils, 12 of them will likely be replaced with IE fat made with palm oil or fully hydrogenated soybean oil. Approximately fifty percent of the total *trans* fat intake in American diet came from these 12 food categories, with cakes, cookies and other baked goods being the predominant dietary sources of *trans* fat. However, the amount of total palmitic and stearic acid that comes from the IE fat as an alternative fat for TFA-containing oil is unknown.

Chemical interesterification (CIE) modifies the fat triacylglycerol structure by rearranging the fatty acids at positions *sn*-1, -2, and -3 of the glycerol backbone, and at the same time, a higher proportion of SFAs end up at the *sn*-2 position [6]. Although interesterified fats (IE) with their flexibility in physical and functional characteristics are beneficial for food manufacturing purposes, the health implications of long term consumption, mainly on insulin resistance, serum lipids, and adiposity are still inconclusive [3].

A 6-h postprandial study by Berry et al. involving 20 healthy men found that interesterified palm oil did not result in adverse changes in postprandial plasma insulin compared to native palm oil [7]. On the other hand, Yli-Jokipii et al. studied ten healthy women over six hours after an oral fat load, and showed that the natural palm oil induced significantly lower insulin secretion compared to interesterified palm oil with abundance of palmitic acid [8]. In longer-term study, a 4-week human feeding trial of 30 volunteers by Sundram et al. showed that a stearic acid-rich interesterified fat lowered fasting and postprandial insulin concentrations compared to palm olein [9]. However, a subsequent study with 41 subjects by Filippou et al. was not able to duplicate the results reported by Sundram et al. [10], where the study observed no difference in insulin secretion or postprandial glucose changes with 6-weeks of each treatment phase in comparison to the interesterified palm olein with native palm olein.

In comparisons of diets rich in IE fat to diets rich in native fat with similar fatty acid compositions, many studies have demonstrated that diets rich in IE fat, which contain more triacylglycerol (TAG) molecules bearing saturated fatty acids (SFAs) in the *sn*-2 position, have a positive influence on postprandial lipemia and are associated with a reduction in postprandial TAG [7,10,11,12]. In several studies carried out over longer periods (18–42 days), no significant differences in fasting serum lipoprotein were found when the consumption of an IE fat-rich diet was compared with a native fat-rich diet [10,13,14].

Similarly, a comprehensive literature review by Baumgartner and Mensink [15], reported that overall, IE fat does not adversely affect serum lipid, plasma glucose, and insulin concentrations. In a more recent review, Mensink et al. concluded that although IE fat has not been associated with any health issues, the potential health risk of longer-term consumption and effects on modifying certain aspects of lipid and glucose metabolism, inflammatory responses, hemostatic parameters, and satiety is of concern [3].

Gouk et al.’s recent studies [16,17] added another exciting piece to the jigsaw puzzle when they showed that C57BL/6 mice fed dietary fats with a preponderance of long chain saturated fatty acids (palmitic acid, stearic acid) at *sn*-1, 3 positions for 15 weeks reduced the deposition of subcutaneous and visceral fat. This result suggested that the positional distribution of fatty acid in TAG molecules may play a role in adiposity.

We aim to investigate the effects of IE fats rich in palmitic acid or stearic acid obtained from interesterified palm olein (IEPalm) and interesterified vegetable oils blend (IEStear), respectively, on serum lipids, surrogate biomarkers of insulin resistance, and adiposity compared to native palm olein (NatPO). We hypothesize that the effects of IE fat diets on serum lipids, apolipoprotein concentrations, and surrogate biomarkers of insulin resistance and adiposity will be different from those of a native palm olein diet.

## 2. Materials and Methods

### 2.1. Subjects

Initially, 154 interested individuals over 20 years of age from the Universiti Putra Malaysia (UPM) community in Serdang, Selangor underwent health screening which included measurement of differential white blood cell count, serum lipid profiling, liver and kidney function tests, and completion of a medical history questionnaire. Finally, 90 individuals (67 women, 23 men) were selected based on the following inclusion criteria: overweight and healthy Malaysian males and females, aged 20–60 years, with body mass index (BMI) scores of 21 to 30 kg/m^2^. The exclusion criteria excluded subjects with blood pressure reading 140/90 mmHg or more, current cigarette smokers, pregnant or lactating individuals, those with plans to go abroad during the study period, fasting serum total cholesterol (TC) > 6.5 mmol/L, fasting serum TAG > 2.0 mmol/L, the presence of two or more times the upper normal limits of alanine- and aspartate-transaminases, plasma creatinine >150 µmol/L, the presence of cardiovascular diseases, diabetes mellitus, hypertension, cancer, or stomach ulcers, or those on lipid or blood pressure-lowering medication/supplements.

The study was conducted in accordance with the Declaration of Helsinki. The protocol was approved by the Joint Research and Ethics Committee of the International Medical University (IMU), Kuala Lumpur (*IMU R104/2012)* and registered in the local institution registry coded PD174/13 before the first patient enrolment in April 2013. The randomized control trial were then registered in the clinicaltrials.gov as *NCT02192047* (URL: https://clinicaltrials.gov/show/NCT02192047) after the study was commenced. Written informed consent was obtained from all subjects.

### 2.2. Test Fats

All oils and fats were obtained from Pasir Gudang Edible Oil Sdn. Bhd. (Johor, Malaysia). The C16:0-rich fats were refined, bleached and deodorized (RBD) palm olein IV 64 (NatPO) and RBD chemically-interesterified palm olein (IEPalm). The C18:0-rich IE vegetable oil blend (IEStear) was prepared by blending fully hydrogenated soybean oil, high oleic acid sunflower oil, and sunflower oil following the chemical interesterification process. The fatty acid compositions of the test fats are shown in Table 1. From these test fats, margarines were produced with a fat content of 80% (*w/w*) in the margarine plant of the Malaysian Palm Oil Board (MPOB), Bangi, Malaysia. The fatty acid compositions of test fats were determined by gas-liquid chromatography**.** Regiospecific analyses of oils were performed using a JEOL ECA-500MHz NMR spectrometer (JEOL Ltd., Tokyo, Japan).

### 2.3. Test Diets

The margarines prepared with test fats were used to bake cupcakes with a margarine content of 33% *w/w* per cake and cookies with a margarine content of 30% *w/w* per piece. Test snacks were packaged individually with two cupcakes per pack and five pieces of cookies per pack. Products were color-coded as blue, red, or green to blind both investigators and subjects.

Each test snack was distributed with a prescribed palm-based background diet, consisting of breakfast, lunch and dinner prepared with RBD palm olein IV56 on weekdays. Test snacks were also provided on every Friday evenings for weekend consumption. A proximate analysis of the energy intakes from the test snacks, background diet, and the total energy from test snacks plus the background diet are shown in Table 2. The background diet and test snack provided ~9.62 MJ energy per day and contained similar proportions of energy from fats (~35% of energy), carbohydrates (~53% of energy), and protein (~12% of energy). The test fats contributed ~20% of energy to the total diet (20.6 ± 0.61% energy/day for NatPO diet; 20.9 ± 0.27% energy/day for the IEPalm diet; 20.59 ± 0.76% energy/day for the IEStear -diet).

### 2.4. Study Protocol

**Figure 1:**
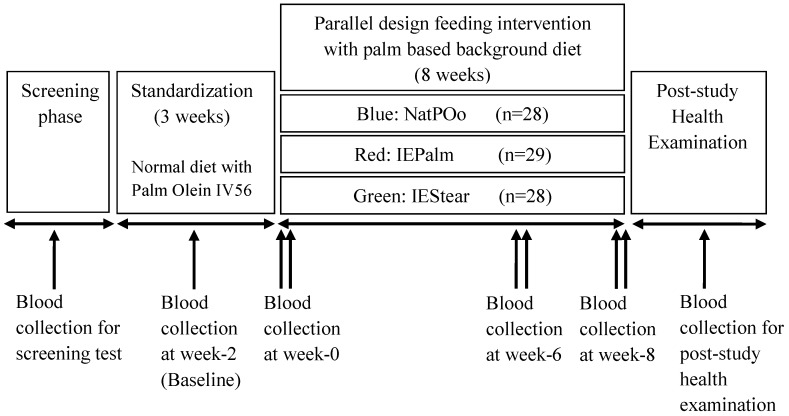
Outline of study design.

A double-blind, parallel design involving 8 weeks of feeding was used. This feeding trial was conducted from 17 December 2013 until 12 March 2014. All subjects received a palm-based standardization diet for three weeks, followed by random assignment to one of the three test fats which were incorporated into snacks and provided daily, together with a palm-based background diet for eight weeks. Each subject was randomly stratified into one of the three dietary groups according to the baseline characteristics that were recorded at week-2 of the standardization period. In other words, the subjects were stratified based on gender and the clinical characteristics (i) fasting serum c-peptide level and (ii) lipid profile including low density lipoprotein cholesterol (LDL-C), high density lipoprotein cholesterol (HDL-C) and TAG, to ensure that there were no differences (*p* < 0.05) across the three diet groups before the commencement of the feeding intervention. During the eight weeks of the feeding intervention, each subject received an assigned test snack for morning breakfast (two test cupcakes) and afternoon tea (five pieces test cookies), which provided a total of 3.8 MJ daily. A background diet prepared with palm olein IV56 was served together with test snacks on weekdays providing 15% of the energy intake from fats. During the weekend, subjects were provided with test snacks and cooking oil. Subjects prepared their meals at home with cooking oil according to the dietary guidelines provided. Subjects recorded the daily consumption of food on weekends and leftover food in a food diary. Subjects were also interviewed individually regarding their time spent on physical activity in past 24 h at the beginning of the study intervention. Physical activity level (PAL) assessments were conducted using the T & Z Calorie Counter [18]. The T & Z Calorie counter was modified from the spreadsheet reported by Gerrior et al. (2006) [19] which has been validated. Each subject was categorised into one of tfour physical activity levels: sedentary (PAL < 1.40), low active (1.4 ≤ PAL < 1.60), moderately active (1.60 ≤ PAL ≤ 1.75), or high active (PAL > 1.75). Anthropometry, blood pressure measurements, and blood samples were collected for two days in a row at week 0, week 6 and at the end of week 8. The outline of the study protocol is shown in Figure 1.

### 2.5. Sample Size Calculation

The required sample size for the study was calculated using fasting serum c-peptide as a primary outcome as proposed in a study by Filippou et al. [10]. It was reported that c-peptide was more superior and stable compared to other insulin-derived measures of insulin resistance in nondiabetic adults [20]. By referring to Filippou et al. and Sundram et al., the average SD (s) of the differences for fasting serum c-peptide was estimated to be 25% [9,10]. At an alpha value of 0.05, a test power of 80%, and a minimum difference in means (d) compared to be clinically significant as 20%., the minimum sample size per group based on Lehr’s equation was calculated to be 16/(d/s)2 =16/(20/25)2, that is, 25. In calculating this minimum required sample size, we considered the two interesterified palm oil diets to be primary treatment groups with the natural palm oil diet being the control group. If all three diets were regarded as treatments, then the approximate sample size needed would be 22/(20/25)2, that is, 35. Since the sample size approximation is conservative, and due to budget constraints, we opted to use the minimum sample size of 25.

### 2.6. Collection and Handling of Blood Samples

Blood collection and anthropometric measures (body weight, BMI, waist circumference) were measured with standard anthropometric tools, and blood pressure (OMRON HEM-7203), basal metabolic rate (BMR) and body composition (body fat percentage, visceral fat) were determined by the OMRON body analyzer (model HBF-356, Europe) at three time points: week 0 (baseline), week 6 and week 8. On each blood sample collection day, 10 mL of fasting venous blood was collected after an overnight fast from 22:00 h and dispensed into appropriate vacutainers (Becton Dickinson, Plymouth, UK). Blood samples were centrifuged at 3000 rpm for 15 min at 4 °C, and the sera was harvested. Oxalated blood was analysed for blood glucose, while sera was analysed for TC, HDL-C, LDL-C, the TC: HDL ratio, lipoprotein (a) [Lp(a)], apolipoproteins A1 (ApoA1) and B100 (ApoB100), TAG, and leptin.

### 2.7. Dietary Compliance

Each day during the distribution of test snacks at the research station, subjects were asked if they any problems had occurred regarding the consumption of the test snacks and the packed meals. Any negative feedback was recorded. A daily “attendance list” was recorded for all participants for their collection of test snacks and meal packs at the station. A subject leader in each of the three groups assisted in the record of attendance and feedback from subjects. 

### 2.8. Statistical Analysis

The data are reported as means ± standard deviations. The statistical analysis of the data was carried out using IBM SPSS Statistic version 20 and GraphPad Prism (version 5.0; GraphPad Software, Inc., San Diego, CA, USA) to compare the changes of outcome measures at week-6 and week-8 from week-0 across the three diets. To test for differences among the three groups, we used one-way ANOVA tests. To test for normality, the Shapiro–Wilk test was applied to the data and it was found that eight measured variables were highly skewed due to the presence of a few outlying values and therefore, were not normally distributed. As a result, transformation of the data to achieve approximate normality would not help. However, a test of homogeneity of variance between the test-diet groups showed that at a 0.05 significance level, the variances could be regarded as similar for the three groups. Due to the non-normality and difference in distributions of the variables, we chose the nonparametric Kruskal–Wallis test and pairwise multiple comparison test with Bonferroni correction. The analysis was repeated on the average of changes for both week 6 and week 8 from week 0. A value of *p* < 0.05 was used to show significance for all tests. The overall *p*-values after the Kruskal–Wallis test for each variable are shown in Table S1. To gain further insight, especially to account for differences in the subjects and comparison of diets, a statistical analysis based on the mixed effect model was conducted, since data were available at the three time points: week 0, week 6 and week 8. The R function lmer in the package lme4 was used. The detection of outliers and analysis of residuals for normality and homogeneity of variances were also carried out using the standard residual plots. In the mixed effect model, random effects due to subjects were considered and the variables were diets and time and the interaction between diet and time. Age and gender were initially included but found to be insignificant. The NatPO diet was used as the reference level for the variable diets in the mixed effect regression analysis. The analyses were repeated by log transforming the dependent variable. A few outliers were detected in the data in the residual analysis. Since they did not significantly affect the results of the analyses, these outliers were not excluded. For the mixed effect regression analysis, the log-transformation greatly improved the normality and homogeneity of variance of the residuals, in particular, for serum insulin and c-peptide. For the subject random effect, the variances were less than 1. This showed that the variations due to subjects were insignificant.

## 3. Results

**Figure 2:**
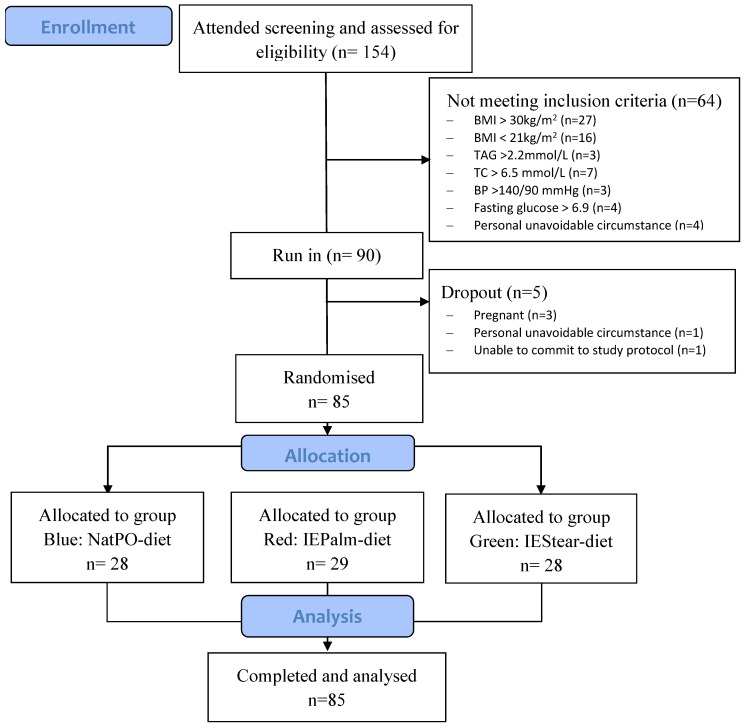
CONSORT flow diagram of subjects throughout the study.

Out of the 90 subjects who participated, a total of 85 (women = 64, men = 21) subjects completed the study. The baseline characteristics of subjects that were recorded at week 2 of the standardization period before the subjects were randomly stratified into three dietary groups are shown in Table 3. Before the run-in (standardization period), 64 subjects did not meet the inclusion criteria and were excluded. During the study, five subjects withdrew due to the reasons shown in the CONSORT diagram (Figure 2). Since the minimum of 25 subjects per group was met, this aspect did not compromise the power of the study design.

The mean BMIs of subjects in the NatPO, IEPalm and IEStear groups were 26.7 ± 2.4, 25.7 ± 2.2, and 26.0 ± 2.7 kg/m^2^ respectively. About half or more of the subjects were overweight but apparently healthy, as determined by the health screening prior to the selection. From the communication with subjects during the distribution of food in the morning, no subject reported that they had any problem with consuming the test snacks.

The primary time point of the present study was week 8. However, outcomes were also measured at week 6 to serve as a comparison with other shorter-period studies of only 6 weeks. Meanwhile, the fasting week 0 samples were taken on the first day of the dietary intervention after grouping. Therefore, we report the results of week 0, week 6, and week 8 in Table 4 and Table 5. The comparisons of changes between each diet groups were made using the changes in week 6 and week 8 from week 0.

**Figure 3:**
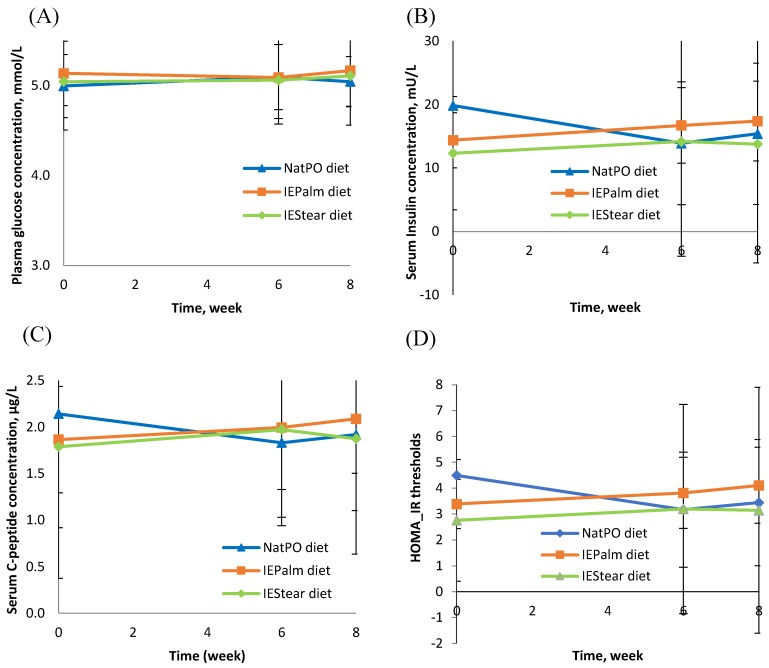
Concentrations of fasting plasma glucose (**A**), serum insulin (**B**), serum c-peptide (**C**), and HOMA-IR threshold after eight weeks feeding intervention among three diet group (NatPO diet, *n* = 28; IEPalm diet, *n* = 29; IEStear-diet, *n* = 28). The values represent means ± SDs at week 0, week 6, and week 8. HOMA-IR, Homeostasis Model Assessment.

There were no significant changes in the fasting state of plasma glucose, serum insulin, or c-peptide concentration between the three diet groups after 8 weeks of the intervention. (Figure 3A–C). Only C-peptide showed some changes over time for the IEPalm diet. These findings were consistent with the finding that there were no significant differences for the markers of insulin resistance (Homeostasis Model Assessment [HOMA-IR]) (Figure 3D) and insulin sensitivity (Quantitative Insulin-Sensitivity Check Index [QUICKI]) among the three test fats.

Changes in serum TAG concentrations from week 0 to the end of week 6, and week 8 were significantly lower in the IEStear diet compared to IEPalm diet (week 6, *p* = 0.035; week 8, *p* = 0.007). However, no differences were found between the NatPO-diet and the other two IE diets. The analyses from the linear mixed effect model supported these conclusions. No significant differences were observed for the changes between the three test diets for the TC, LDL-C, HDL-C, Lp(a), ApoA1 and ApoB100 concentrations. However, IEPalm tend to raise the TC:HDL-C ratio compared to the NatPO and IEStear diets but this was not statistically significant (Table 5).

At the end of week 6, the changes in body weight from week 0 in subjects consuming the NatPO (*p* = 0.024) and IEStear diets (*p* = 0.049) were found to be significantly lower compared with those of subjects on the IEPalm diet. The BMIs of subjects on the NatPO (*p* = 0.016) and IEStear diets (*p* = 0.025) also had significantly lower changes compared to those of subjects on the IEPalm diet. The magnitudes of the changes in body weight and BMI were observed to be in the following order: NatPO < IEStear < IEPalm. This observation suggests that native palm olein may inhibit weight gain compared to interesterified IEPalm. Compared to NatPO diet, the effects of the IEPalm diet on the changes in body weight and BMI were found to be higher but not significant at week 8. Individuals consuming the IEStear diet did not show any differences in body weight or BMI at the end of week 8. The NatPO-diet was also found to have significantly lower changes in body fat percentage (*p* = 0.026) and leptin concentration (*p* = 0.025) compared to the IEPalm diet at the end of week 8 but not compared to the IEStear diet. In the mixed effect model analysis, the slope of body fat percentage against the time variable for NatPO diet was negative. This was fairly significant if age and gender were not taken into consideration.

There was no significant change in the blood pressure, waist-to-hip ratio, or visceral fat level between the three groups after eight weeks. The subjects in the three diet groups were found to be at a moderately active physical activity level (NatPO: 1.73 ± 0.27; IEPalm: 1.73 ± 0.24; IEStear: 1.65 ± 0.29). Therefore, the three groups of volunteers did not differ in their physical activity levels. The results of changes in anthropometry measurements at week 6 and 8 from week 0 are shown in Table 5.

## 4. Discussion

Our findings indicate that both IEPalm and IEStear have similar effects to NatPO on surrogate markers of insulin resistance. These results agree with the findings of Filippou et al., where the release of insulin and glucose was not affected by the replacement of NatPO (*sn*-2: C16:0 = 9.8 mol%) with that of IEPalm (*sn*-2: C16:0 = 45.9 mol%) [10]. However, our results do not support those of Sundram et al. [9] who reported that an interesterified high stearic acid diet adversely affected glucose metabolism relative to an native saturated fat diet in healthy humans. It is pertinent to point out that the amount of C18:0 in the diet of Sundram et al.’s study [9] was 50% higher (12.5% en) than that of the IEStear diet (8.1% en) in the present study which might have contributed to these different outcomes from the two studies. We suggest that the surrogate markers of insulin resistance were not affected by the positional distribution of fatty acids in TAG molecules or the types of SFAs (C16:0 and C18:0) dominant in the diets.

Overall, the two IE test fats (IEPalm and IEStear), when compared with the control (NatPO), did not show any significant difference in TC, HDL-c, LDL-c, TC/HDL-c, Lp(a), ApoA-1, ApoB-100 after eight weeks. However, it is noteworthy that changes in serum TAG levels in the IEPalm group were significantly higher in the IEStear group, although the changes in both IE fat groups were not significantly different to the control NatPO group. The C18:0-rich IEStear diet lowered serum TAG marginally when compared with the 16:0-rich native NatPO diet, but significantly when compared with the 16:0-rich IEPalm diet. Our eight weeks intervention results for serum TAG compared with native NatPO and IEPalm are similar to the findings of Filippou et al., who reported no significant difference in serum TAG concentrations between groups consuming native palm olein versus interesterified palm olein in their six-week study [10].

The increase in the serum TAG might arise from a greater synthesis of hepatic VLDL which is secreted into the circulation [21]. A higher serum TAG concentration will lead to a greater fat deposition (lipogenesis) in adipose tissue [22]. This explanation was supported by the increases in body weight and BMI at week 6 and percentage body fat at week 8 in the IEPalm diet group, which also had a higher serum TAG than the other diet groups. However, these changes in weight and BMI were not observed at the end of week 8, probably due to the control action of leptin, an indispensable hormone in the physiological control of energy balance [23]. We propose that a new steady state was reached at week 6, whereby significantly higher changes in body weight and BMI were observed in subjects on the IEPalm diet. Although these anthropometric indices were still elevated at week 8, they did not reach statistical significant difference compared to week 0. Support for this new steady state interpretation is the significantly increase in serum leptin levels at weeks 6 and 8 in the IEPalm diet compared to the NatPO diet. The increase in percentage body fat demonstrated that increase in the serum leptin level with the increase in fat deposition will elevate the synthesis of leptin by adipocytes, causing the serum leptin to rise; this was seen in the subjects on the IEPalm diet in the present study.

The leftover meal packs were recorded daily by the subjects, and our examination of this data from the beginning to the end of intervention did not reveal any differences. This removes the bias that any weight gain at week 6 and week 8 was due to greater food consumption compared with week 0.

The present study also suggested that different fatty acids situated predominantly at the *sn*-2 position, namely C18:1, C18:0 and C16:0 in NatPO, IEStear, and IEPalm, might influence weight gain and adiposity. Although the level of physical activity determined by T& Z calorie counter was not significantly different across the three test groups, we found that changes in weight and BMI at week 6 with IEStear and NatPO diets were significantly lowered compared with those of individuals on the IEPalm diet. Therefore, in line with the study carried out by Gouk et al. which demonstrated that the feeding of dietary fats with preponderance long chain saturated fatty acid at *sn*-1,3 position reduces fat deposition in the mice model [16]. In the present study, we found that when more SFA (C16:0) is present at the *sn*-1, 3 position on the TAG backbone of the fat in NatPO, there is significantly lower weight gain and changes in BMI compared to the lesser SFA (C16:0) situated at the *sn*-1, 3 position of the fat in IEPalm. On top of that, based on the length of the SFA located at the *sn*-1, 3 position, as explained by Gouk et al. [17], the longer chain SFA (C18:0) present at the *sn*-1, 3 in IEStear had lower changes in body weight gain and BMI compared to the comparable amount of shorter chain SFA (C16:0) from IEPalm.

Interestingly, there was a marginal rise of serum TAG at week 6 in the IEPalm diet, which significantly increased at week 8. This could be due to an elevated rate of adipose tissue lipogenesis for the same period [21]. Specifically, the IEPalm with a higher concentration of C16:0 located at the *sn*-2 position of the TAG backbone tended to raise serum TAG, contributing to body weight gain, an increase in body fat percentage, a rise in circulating leptin, and hence, an increased risk of obesity. This serum TAG-raising effect was not seen when C18:0 and oleic acid (C18:1) occupied the *sn*-2 position in IEStear and natural NatPO, respectively. In other words, compared to C18:0 and C18:1, the location of C16:0 at the *sn*-2 position of the triglyceride backbone might adversely affect the body by inducing serum triglyceride levels that play a role in increasing the risk of obesity.

To our knowledge, the present study with its eight-week intervention period reported longer term effects of IE fats on healthy overweight subjects. Overweight subject were selected because individuals with higher BMI scores have an increased risk of hyperinsulinemia that could potentially lead to weight gain [24]. Besides this, we are the first to show the adverse effects of IE fats on serum leptin levels and adiposity. In non-obese people, circulating leptin concentrations are directly proportional to fat deposition and therefore, an increase in the fat mass of adipose tissue [23]. This suggests that IE fat (IEPalm) contributes to an increased in body fat deposition; this was substantiated by the increased in percentage body fat data collected. However, the results in the present study only refer to IE fats that are formulated as shown in Table 1, as different effects may be obtained depending on the fatty acid compositions or the process of preparation of the test fats.

In the comparison of the effects of C16:0- and C18:0-rich diets on the lipid profile, an earlier study by Hayes and Khosla suggested that palmitic acid (C16:0) is not hypercholesterolemic when given in conjunction with dietary cholesterol levels [25]. However, Schwab et al. observed that diets rich in C16:0 raise TC and LDL-C levels compared to diets rich in stearic acid [26]. This is in line with the raising trend of the changes in TC and LDL-C after the consumption of palmitic acid-rich diets (NatPO, IEPalm) versus a stearic acid-rich diet (IEStear) that are shown in the current findings. However, both were not significant. Compared to the stearic acid-rich IEStear diet, the NatPO diet showed a similar effect on the serum lipoprotein level, while the IEPalm diet was found to raise TC:HDL-C, but this was not statistically significant. Therefore, we suggest that palmitic acid from the native NatPO diet is as neutral as stearic acid from the IEStear diet in terms of the cholesterolemic response, but the cholesterolemic effect of chemically modified palmitic-rich fat (IEPalm) still needs further investigation.

Among c-peptide, insulin, and glucose, c-peptide was found to be the most stable biomarker. However, c-peptide by itself could not represent the insulin resistance calculated using the HOMA-IR equation. Thus, a limitation of this study is that c-peptide was chosen as the primary outcome. It was chosen because of its stability and measurability compared to the calculated HOMA-IR. Another limitation is that this study was represented by the preponderance of female over male subjects. However, we did assign them equally based on gender to the three diet groups. Most of the subjects in our study were of Malay ethnicity, hence not representing the multi-ethnic general Malaysian population. However, the study was not designed to investigate the effects of ethnicity on the outcomes concerned. Another limitation was that we did not have an objective measure for dietary compliance.

## 5. Conclusions

We conclude that both interesterified test fats—IEPalm (rich in 16:0) and IEStear (rich in 18:0)—had no significant effects on insulin resistance. However, the IEPalm diet marginally induced adverse effects by raising body weight and BMI at week 6 and serum TAG, body fat percentage, and leptin concentrations at week 8. We postulate these effects could have been due to greater fat absorption and lipogenesis in adipose tissue for the IEPalm group, which suggests that the types and lengths (C16:0 and C18:0) of the fatty acids predominantly situated on the TAG molecule play an important influence on lipid metabolism.

## Figures and Tables

**Table 1:** Fatty acid compositions of the three test fats.

Fatty Acid Composition of Test Fat (%)	NatPO		IEPalm		IEStear	
	Mean	SD	Mean	SD	Mean	SD
C12:0						
Total	0.41	0.04	0.64	0.03	0.08	0.01
*sn*-2	0.54	0.03	0.64	0.09	0.52	0.30
C14:0						
Total	1.08	0.08	1.24	0.03	0.12	0.01
*sn*-2	0.36	0.31	1.19	0.12	ND	
C16:0						
Total	37.59	3.77	35.02	0.60	7.83	0.09
*sn*-2	11.11	0.82	32.42	4.02	17.86	5.58
C18:0						
Total	4.14	0.35	5.08	0.11	39.28	0.69
*sn*-2	0.96	0.15	3.98	0.47	26.72	5.27
C18:1						
Total	43.99	2.91	44.83	0.02	41.19	0.47
*sn*-2	63.71	1.56	48.98	4.46	43.09	1.13
C18:2						
Total	12.17	1.09	12.66	0.38	10.93	0.18
*sn*-2	23.01	0.84	12.44	0.88	10.68	0.22
C18:3						
Total	0.23	0.02	0.13	0.02	0.04	0.07
*sn*-2	0.31	0.18	0.04	0.06	ND	
C20:0						
Total	0.39	0.01	0.40	0.03	0.47	0.01
*sn*-2	ND		0.26	0.05	ND	
Others						
Total	0.01	0.01	0.01	0.01	0.07	0.06
*sn*-2	0.00		0.06	0.07	1.13	1.22
**Total**						
SFA	43.6	4.0	42.4	0.4	47.8	0.7
MUFA	44.0	2.9	44.8	0.1	41.2	0.5
PUFA	12.4	1.1	12.8	0.4	11.0	0.3

SFA, saturated fatty acid; MUFA, monounsaturated fatty acid; PUFA, polyunsaturated fatty acid; ND, non-detectable; NatPO, native palm olein; IEPalm, interesterified palm olein; IEStear, interesterified vegetable oil blend.

**Table 2:** Daily dietary intake of subjects in 8 weeks intervention.

	NatPO		IEPalm		IEStear	
	Mean	SD	Mean	SD	Mean	SD
(a) Energy of test snack (kcal)	912.2	22.7	913.6	5.3	910.3	11.8
Fat (kcal)	489.1	19.4	494.3	7.0	486.4	19.9
SFA (kcal)	213.3	19.6	209.5	2.0	232.3	3.2
MUFA (kcal)	215.1	14.2	221.6	0.1	200.3	2.3
PUFA (kcal)	60.6	5.4	63.2	2.0	53.4	1.2
(b) Energy of background diet (kcal)			1451.7	1.5 *		
Fat (kcal)			354.4	0.6 *		
SFA (kcal)			161.3	0.05 *		
MUFA (kcal)			150.02	0.17 *		
PUFA (kcal)			43.05	0.13 *		
(c) Test snack + background diet						
Total energy (kcal)	2364.0	23.5	2365.3	4.1	2362.0	10.05
Total fat (% en)	35.7	0.5	35.9	0.3	35.6	0.7
Total protein (% en)	12.1	0.1	12.2	0.1	12.2	0.1
Total carbohydrates (% en)	52.2	0.4	52.0	0.2	52.2	0.6

SFA, saturated fatty acid; MUFA, monounsaturated fatty acid; PUFA, polyunsaturated fatty acid. * The value is similar for all three experimental groups.

**Table 3:** Characteristics of each group after two weeks of run in (women = 64, men = 21).

Characteristic/Parameter	NatPO (Women = 21, Men = 7)		IEPalm (Women = 22, Men = 7)		IEStear (Women = 21, Men = 7)		Normal Range
	Mean	SD	Mean	SD	Mean	SD	
Age (years)	31.3	5.4	37.1	9.8	34.8	8.7	NA
Height (cm)	157.6	6.3	158.8	9.7	159.2	6.5	NA
Weight (kg)	66.1	8.1	65.9	11.2	65.8	9.2	NA
Waist (cm)	83.1	8.3	81.6	8.9	84.4	7.9	Women <80, Men <90
BMI (kg/m^2^)	26.7	2.4	25.7	2.2	26.0	2.7	17.5 to 22.9
Visceral fat level *	8	3	8	2	8	4	0–9
Body fat composition (%)	32.5	4.4	32.9	5.1	32.5	4.3	Women <30, Men <20
BMR (kcal)	1351	149	1330	204	1346	146	NA
Systolic blood pressure (mm Hg)	113.3	11.1	116.2	10.9	113.2	12.1	<120
Diastolic blood pressure (mm Hg)	73.1	8.1	75.0	7.6	73.8	7.6	<80
Serum TC (mmol/L)	4.95	0.64	5.07	0.85	4.96	0.83	<5.2
Serum HDL-C (mmol/L)	1.39	0.27	1.41	0.28	1.36	0.26	>1.0
Serum LDL-C (mmol/L)	2.98	0.60	3.12	0.70	3.09	0.75	<2.6
Serum TAG (mmol/L)	1.28	0.85	1.20	0.60	1.13	0.56	<1.7
Fasting plasma glucose (mmol/L)	4.95	0.45	4.95	0.55	4.94	0.36	3.9–5.5
Fasting serum insulin (mU/L)	10.7	6.4	11.2	9.3	10.5	6.5	<25
Fasting serum C-peptide (μg/L)	1.67	0.64	1.66	0.73	1.66	0.59	0.5–2.0

BMI, Body mass index; BMR, basal metabolic rate; TC, total cholesterol; HDL-C, high density lipoprotein cholesterol; LDL-C, low density lipoprotein cholesterol; TAG, triacylglycerol. * Visceral fat level classification: 1–9 as normal, 10–14 as high, 15–30 as very high (OMRON healthcare figures).

**Table 4:** Means ± SDs of surrogate markers measured at week 0, week 6 and week 8.

	NatPO Diet			IEPalm Diet			IEStear Diet		
	Week 0	Week 6	Week 8	Week 0	Week 6	Week 8	Week 0	Week 6	Week 8
Glucose (mmol/L)	5.00 ± 0.35	5.10 ± 0.36	5.04 ± 0.28	5.14 ± 0.54	5.09 ± 0.49	5.17 ± 0.55	5.04 ± 0.36	5.06 ± 0.46	5.11 ± 0.40
Insulin (mU/L)	19.85 ± 34.67	13.91 ± 9.67	15.40 ± 11.13	14.38 ± 8.93	16.72 ± 18.07	17.39 ± 18.72	12.34 ± 4.36	14.18 ± 5.96	13.78 ± 6.28
C-peptide (μg/L)	2.14 ± 1.77	1.83 ± 0.80	1.92 ± 0.82	1.87 ± 0.87	2.00 ± 1.03	2.09 ± 1.24	1.79 ± 0.57	1.97 ± 0.67	1.88 ± 0.59
HOMA-IR	4.50 ± 8.04	3.17 ± 2.22	3.44 ± 2.44	3.39 ± 2.35	3.82 ± 4.04	4.11 ± 4.75	2.76 ± 0.96	3.20 ± 1.37	3.14 ± 1.47
QUICKI	0.33 ± 0.03	0.33 ± 0.02	0.33 ± 0.02	0.33 ± 0.03	0.33 ± 0.03	0.33 ± 0.03	0.33 ± 0.02	0.33 ± 0.02	0.33 ± 0.02
TC (mmol/L)	5.00 ± 0.72	5.06 ± 0.72	5.19 ± 0.57	5.26 ± 0.92	5.39 ± 0.85	5.46 ± 0.81	5.14 ± 0.60	5.11 ± 0.67	5.30 ± 0.70
LDL-C (mmol/L)	3.13 ± 0.65	3.18 ± 0.62	3.20 ± 0.57	3.37 ± 0.78	3.45 ± 0.69	3.44 ± 0.64	3.26 ± 0.56	3.26 ± 0.60	3.39 ± 0.70
HDL-C (mmol/L)	1.31 ± 0.26	1.32 ± 0.27	1.36 ± 0.27	1.40 ± 0.32	1.41 ± 0.28	1.40 ± 0.30	1.33 ± 0.24	1.33 ± 0.21	1.39 ± 0.24
TC:HDL ratio	3.95 ± 0.92	3.99 ± 0.93	3.94 ± 0.81	3.89 ± 0.89	3.94 ± 0.77	4.03 ± 0.81	3.98 ± 0.80	3.92 ± 0.79	3.91 ± 0.78
Lipoprotein (a) (mg/dL)	17.91 ± 16.43	17.32 ± 16.14	16.54 ± 14.67	18.22 ± 14.16	17.24 ± 10.90	17.98 ± 12.94	18.30 ± 14.10	17.98 ± 13.79	17.52 ± 13.62
Apo-A1 (g/L)	1.46 ± 0.17	−0.004 ± 0.12	0.04 ± 0.13	1.52 ± 0.23	0.02 ± 0.12	0.04 ± 0.13	1.48 ± 0.14	0.002 ± 0.10	0.05 ± 0.10
Apo-B100 (g/L)	1.05 ± 0.20	1.05 ± 0.20	1.07 ± 0.17	1.11 ± 0.24	1.13 ± 0.23	1.15 ± 0.21	1.07 ± 0.17	1.06 ± 0.18	1.09 ± 0.19
TAG (mmol/L)	1.25 ± 0.64	1.23 ^a,b^ ± 0.69	1.39 ^a,b^ ± 0.87	1.07 ± 0.37	1.21 ^a^ ± 0.54	1.36 ^a^ ± 0.69	1.21 ± 0.66	1.12 ^b^ ± 0.65	1.16 ^b^ ± 0.80
Leptin (ng/mL)	19.49 ± 14.67	20.16 ± 15.19	19.58 ^a^ ± 14.58	16.890 ± 11.53	18.96 ± 12.12	19.29 ^b^ ± 9.96	16.94 ± 6.91	18.19 ± 8.01	17.76 ^a,b^ ± 6.78

TC, total cholesterol; HDL-C, high density lipoprotein cholesterol; LDL-C, low density lipoprotein cholesterol; HDL-C, high density lipoprotein cholesterol; TAG, triacylglycerol; Apo-A1, apolipoprotein A1; Apo-B100, apolipoprotein B100. ^a,b^ Different superscript letters in the same rows indicate that the mean values showed statistically significant differences (*p* < 0.05, Kruskal–Wallis multiple comparison test) between corresponding columns.

**Table 5:** Means ± SDs of surrogate markers measured at week 0, week 6 and week 8.

		NatPO Diet			IEPalm Diet			IEStear Diet	
		Changes from Week 0			Changes from Week 0			Changes from Week 0	
	Week 0	Week 6	Week 8	Week 0	Week 6	Week 8	Week 0	Week 6	Week 8
Weight (kg)	66.32 ± 8.00	66.85 ^a^ ± 8.29	66.44 ± 8.13	65.37 ± 10.16	66.79 ^b^ ± 10.33	66.16 ± 10.09	66.09 ± 9.13	66.76 ^a^ ± 9.17	66.54 ± 9.13
BMI (kg/m^2^)	26.65 ± 2.38	26.860 ^a^ ± 2.47	26.70 ± 2.41	25.81 ± 2.21	26.39 ^b^ ± 2.43	26.14 ± 2.36	26.01 ± 2.60	26.27 ^a^ ± 2.58	26.18 ± 2.56
Body fat (%)	32.42 ± 4.43	32.73 ± 4.30	31.84 ^a^ ± 4.72	32.67 ± 5.62	33.02 ± 5.39	32.62 ^b^ ± 5.88	32.34 ± 4.52	32.15 ± 4.33	32.30 ^a,b^ ± 4.01
Visceral fat level ^§^	8.54 ± 2.99	8.64 ± 2.90	8.50 ± 2.96	8.31 ± 2.30	8.66 ± 2.50	8.55 ± 2.52	8.32 ± 3.81	8.54 ± 3.67	8.46 ± 3.55

BMI, Body mass index. ^§^ Visceral fat rating classification: 1–9 as normal, 10–14 as high, 15–30 as very high (OMRON healthcare figures). ^a,b^ Different superscript letters in the same rows indicate that the mean values showed statistically significant differences (*p* < 0.05, Kruskal–Wallis multiple comparison test) among corresponding columns.

## Supplementary Materials

The following are available online at http://www.mdpi.com/2072-6643/10/8/1112/s1, Table S1: Overall *p*-values from the Kruskal–Wallis test for each marker at week 6 and 8.
