# Supplementary material for: Interesterified Palm Olein (IEPalm) and Interesterified Stearic Acid-Rich Fat Blend (IEStear) Have No Adverse Effects on Insulin Resistance: A Randomized Control Trial
Source: Nutrients. 2018 Aug 17;10(8):1112. doi: 10.3390/nu10081112 (PMC6115919; doi:10.3390/nu10081112)
Supplement: Supplementary file 1 [file nutrients-10-01112-s001.zip › Supplemental Table S1.docx]

**Table S1**: Overall *p*-value of Kruskal-Wallis test for each marker at week-6 and -8.

|  | *p*-value | |
| --- | --- | --- |
|  | Week-6 | Week-8 |
| Weight (kg) | 0.013 | 0.183 |
| BMI (kg/m^2^) | 0.007 | 0.132 |
| % Body fat (%) | 0.182 | 0.022 |
| Visceral fat rating | 0.426 | 0.239 |
| Glucose (mmol/L) | 0.479 | 0.557 |
| Insulin (mU/L) | 0.248 | 0.857 |
| C-peptide (μg/L) | 0.474 | 0.194 |
| HOMA-IR | 0.391 | 0.857 |
| QUICKI | 0.090 | 0.960 |
| TC (mmol/L) | 0.491 | 0.857 |
| LDL-C (mmol/L) | 0.802 | 0.491 |
| HDL-C (mmol/L) | 0.501 | 0.392 |
| TC:HDL ratio | 0.228 | 0.143 |
| Lipoprotein (a) (mg/dL) | 0.534 | 0.921 |
| Apo-A1 (g/L) | 0.386 | 0.802 |
| Apo-B100 (g/L) | 0.925 | 0.898 |
| TAG (mmol/L) | 0.033 | 0.009 |
| Leptin (ng/ml) | 0.368 | 0.025 |

BMI, Body metabolic index; HOMA-IR, Homeostasis Model Assessment; QUICKI, Quantitative Insulin-Sensitivity Check Index; TC, total cholesterol; HDL-C, high density lipoprotein cholesterol; LDL-C, low density lipoprotein cholesterol; HDL-C, high density lipoprotein cholesterol; TAG, triacylglycerol; Apo-A1, apolipoprotein A1; Apo-B100, apolipoprotein B100.
